# Supplementary material for: A simple model considering spiking probability during extracellular axon stimulation
Source: PLoS One. 2022 Apr 21;17(4):e0264735. doi: 10.1371/journal.pone.0264735 (PMC9022861; doi:10.1371/journal.pone.0264735)
Supplement: S1 Appendix — (DOCX) [file pone.0264735.s001.docx]

**Appendix**

The relationship between DR and RS.

Let $X\sim N\left( \mu,\sigma^{2} \right)$ then $RS=\frac{\sigma}{\mu}$ and furthermore, the p-quantile of $X$ is that real number $x_{p}$ with

$$P\left( X\leq x_{p} \right)\geq p\wedge P\left( x_{p}\leq X \right)\geq1-p.$$

For continuous distributions this results as a solution of the equation $F\left( x_{p} \right)=p$, which in the case of the normal distribution is reduced by the relation to the standard normal distribution to $p=\Phi\left( \frac{x_{p}-\mu}{\sigma} \right)$, which yields in

$$x_{p}=\sigma\Phi^{-1}\left( p \right)+\mu$$

For $x_{p},x_{q}\in R$ we obtain

$$x_{p}-x_{q}=\sigma\Phi^{-1}\left( p \right)+\mu-\left( \sigma\Phi^{-1}\left( q \right)+\mu\right)=\sigma\left( \Phi^{-1}\left( p \right)-\Phi^{-1}\left( q \right) \right)$$

Therefore

$$\frac{\sigma}{\mu}=\frac{x_{p}-x_{q}}{\mu\left( \Phi^{-1}\left( p \right)-\Phi^{-1}\left( q \right) \right)}$$

For the dynamic range (x_90%_ - x_10%_) defined according to [21], the following applies

$$RS=\frac{\sigma}{\mu}=\frac{x_{90\text{\%}}-x_{10\text{\%}}}{\mu\left( \Phi^{-1}\left( 0.9 \right)-\Phi^{-1}\left( 0.1 \right) \right)}\approx\frac{x_{90\text{\%}}-x_{10\text{\%}}}{2.56\mu\cdot}$$

and the dynamic range normalized by threshold is 2.56 times RS.
