# Supplementary material for: A simple model considering spiking probability during extracellular axon stimulation
Source: PLoS One. 2022 Apr 21;17(4):e0264735. doi: 10.1371/journal.pone.0264735 (PMC9022861; doi:10.1371/journal.pone.0264735)
Supplement: Attachment — Submitted filename: Response to reviewers.docx [file pone.0264735.s002.docx]

Response to the comments of the reviewers

Many thanks for your comments that were very useful to improve the quality and readability of the ms. Please find below your comments in black, our responses in blue and new text in red.

**Reviewer #1:** A simple model considering spiking probability and noise was investigated in the present MS. Due to that too many information were not presented or the descriptions were too professional, I can not judge the importance of the present paper and how the results are acquired. For example, what’s the importance of the three points of the results, and how the results of tables or figures are acquired? And even no a whole model for the nerve fiber is provided. The nerve fiber consists of nodes of Ranvier and internodes were considered. What’s the model for nodes of Ranvier, what’s the model for internode, which were not stated clearly. In general, if the behavior of the nerve fiber is near the threshold, the noise will play important roles in modulating the generation of action potential. Both the membrane potential and noise type and intensity influence the generation of action potential. If the models of the nodes of Ranvier and internode are different, the results are complex due to different thresholds for the Ranvier and internode. In addition, the distance or number of Ranvier are also important. However, the results for the action potentials at nodes of Ranvier and internode, changes of action potential along with the distance, changes of the action potential with noise were not presented.

Many thanks for the critical remarks concerning missing information. We extended the introduction for the non-expert reader, for example the underlined parts are new to be easily to understand.

Experiments and theoretical investigations demonstrate that the axon is the most excitable part of a neuron for stimulation with microelectrodes as used in neuroprosthesis (Porter 1963, Rattay 1999). Although, computer simulations are important tools to improve the generated patterns in the stimulated axons, most of them provide only data about the threshold current needed to generate an AP in a target neuron. However, for a selected axon a second information is important, too. There is an interval of the stimulus intensity where the probability to obtain an AP per stimulating pulse increases from 0 to 1. Unfortunately, quite few modeling studies include a stochastic component in order to replicate the noisiness of recorded spike patterns elicited by electrical stimulation, e. g. in cochlear implants (Hochmair-Desoyer et al. 1984, Rubinstein 1995, Rattay 2000, Badenhorst et al. 2016). Here, we present a simple method to analyze the impact of parameters (e.g. axon type, axon diameter, electrode distance, duration and intensity of the stimulus pulse) on the probability that every stimulus pulse initiate an AP in the axon.

In comparison with a technical cable the conductivity of the intracellular fluid in the axon is very low and, second, the cell membrane is no perfect insulator. These two peculiarities cause a loss of the signal within a distance in the mm range if the signal is not amplified. Two types of axons exist, non-myelinated and myelinated ones. The non-myelinated axon has a uniform membrane with ion channels that enables the conductance of an AP with constant amplitude because the sodium and potassium ion currents across the active membrane compensate current leakage and excite the axon in front of the propagating AP. Myelinated axons have isolating sheaths of lipids around the axon. The myelin layer has short interruptions, called Nodes of Ranvier (NoR), where the conduced AP is amplified by the active cell membrane. As the myelinated ‘internode’ is much longer than the NoR myelinated fibers enjoy three major advantages over non-myelinated ones: (i) the conduction is faster, (ii) less energy is required, and (iii) fast conducting axons are thinner.

Moreover we re-wrote large parts of the method section, added information on our compartment models and presented most model data in a new table
**Table 1. Model parameters and formulas for the opening and closing rates of the ion channels.**
Additionally, Fig. 2 is changed to introduce technical components. It should be clear now that the noisy currents are added to the active membrane compartments and not to the internodes.
**Fig. 2. Compartment model of a myelinated axon.** **A:** Geometry. The active compartments (nodes of Ranvier) are marked in red, the passive ones (internodes) in blue. **B:** Corresponding electrical network model. Independent noise currents $I_{noise,n}$ are added to the active compartments. The stimulating current from the electrode defines the extracellular potential ($V_{e}$) …

Moreover, a new Fig. 4 was added (in exchange to tables) to underline the results
**Fig. 4. RS increases linearly with knoise. A:** Spiking probability functions for 3 knoise values. Numerical evaluation was performed at 100 intensities, from 0.6 threshold to 1.6 ·threshold in 0.01 steps. For each point 500 runs were performed to estimate the spiking probability, then a cumulative Gaussian distribution was fitted. Electrode distance 500μm, myelinated axon d = 1 µm. **B:** RS values from A as functions of knoise underlines the assumption that for small knoise values RS is proportional to knoise. Linear fit: RS = 14458*knoise – 0.588, R² = 0.956.

Why the standard normal distribution?

We added the underlined words in the introduction
After evaluations of experiments Verveen recognized that firing probability as a function of stimulus intensity can be approximated by the cumulative distribution function of the normal distribution and titled the coefficient of variation of the approximating distribution the relative spread …

How the results of tables were acquired?

The problem of the original version was that not enough information was given in the Method section. This is much better now. Moreover, some of the tables were skipped as they did not significantly contribute.

Another sense for the present MS is too “specialized”, which is based on the results of some references. It is difficult to read for most of the readers, due to the contents and the expressions. In summary, the present paper maybe important, but which has not been presented in the abstract, introduction and results. If the MS can not be improved to a very large extent, I do not suggest the MS to be published.

The aim was to present a simple method to simulate the spiking probabilities as seen in stimulation experiments with microelectrodes. We hope we could close the lack of information for the non-expert reader by replacing less essential results with familiarizing parts in the introduction, methods and result section. Moreover, in order to motivate modelers, we added a last sentence in the abstract
The presented method is applicable for other membrane models and can be extended to whole neurons that are described by multicompartment models.
and at the end of the discussion.
While in these cited studies long regular axons are stimulated other modeling work is concerned with non-homogenous axons that include low threshold sodium channels in the axon initial segment of retinal neurons or pyramidal cells (Werginz et al. 2014, Rattay and Wenger 2010). In such studies on low current stimulation the presented method is recommended to obtain more insight about the population of excited cells around an active electrode during microstimulation of the retina or cortex (Kumaravelu et al. 2021).

**Reviewer #2**: The paper looks very raw, like a technical report. Though the underlying investigation is interesting. The main outcome of the paper is that the proposed model well matches the measurements if its parameters are fitted to the data. However, to understand this, the reader has to study the whole manuscript. It is clear that the paper should be reorganized. The main idea should be placed into the abstract and the introduction, the tables should be removed to supplementary and/or replaced by charts, the comparison of the proposed model and existing ones should be made explicitly. After a complete revision of the manuscript it should be studied more carefully for some minor issues.

Many thanks for the critical remarks. We reorganized the whole manuscript and added several explanations because Reviewer 1 criticized that it was not easy to understand for the non-expert. On the other hand, we skipped material (Tables) to reduce reading of non-significant results. **Concerning the main idea**, we proposed a method that should inspire other modelers to simulate stochastic effects seen during extracellular stimulations, but which are missed in the plenty of modeling studies for neural prostheses, e.g. for retinal implants, spinal cord and deep brain stimulation. In order to motivate modelers, we added a last sentence in the abstract The presented method is applicable for other membrane models and can be extended to whole neurons that are described by multicompartment models. and at the end of discussion.
While in these cited studies long regular axons are stimulated other modeling work is concerned with non-homogenous axons that include low threshold sodium channels in the axon initial segment of retinal neurons or pyramidal cells (Werginz et al. 2014, Rattay and Wenger 2010). In such studies on low current stimulation the presented method is recommended to obtain more insight about the population of excited cells around an active electrode during microstimulation of the retina or cortex (Kumaravelu et al. 2021).

**Concerning** “comparison of the proposed model and existing ones should be made explicitly.”
we added a para in the discussion section
Contrary to our simplification of constant RMS amplitudes, the noise term was simulated to increase in an exponential way with transmembrane voltage (Badenhorst 2016), which raises the technical effort. These technical adjustments also showed the negative linear trend between log(RS) to log(diameter) and the values were of similar magnitude as shown in Equ. 11 and Fig. 5. Another approach is to simulate the ion channels individually by Markov processes (Goldwyn et al. 2011) and thus account for the random opening and closing behavior more accurately, whereby there are also gradations in sophistication, ranging from two-rate models to multi-rate models. Works based on stochastic modeling of single sodium channels also showed the decrease of RS with increase of excitable area (Chow and White 1996, White et al. 2000). Their results are qualitatively transferable to our method because the membrane area is directly proportional to the number of sodium channels.
