# Supplementary material for: A simple model considering spiking probability during extracellular axon stimulation
Source: PLoS One. 2022 Apr 21;17(4):e0264735. doi: 10.1371/journal.pone.0264735 (PMC9022861; doi:10.1371/journal.pone.0264735)
Supplement: Attachment — Submitted filename: Response to reviewers.docx [file pone.0264735.s003.docx]

Response to the comments of the reviewers

Many thanks for your comments that were very useful to improve the ms. again.

We have redrawn fig 5 (bad quality) and introduced capitals for first letters in all figures, corrected typing and made few corrections in additions to your suggestions.

Please find below your comments in black, our responses in blue and new text in red.

**Reviewer #1:** The revised MS has been improved to a large extent. I suggest the MS to be published after revisions.
Many thanks for your assessment.

(1) The author claim that they consider an important factor which is often ignore in other investigations. The results acquired with their model close match the experimental results. The author should present the shortage of the results acquired by other models and compare their results and other results. In addition, the significance or potential application of this model or method should be presented.
We added text in the discussion section.
Most of recent modeling work on extracellular nerve stimulation uses a two-step procedure: (i) determination of the electric potential induced by a stimulating electrode in a biological structure and (ii) prediction of its consequences for single-neuron responses (Romeni et al. 2020, Eiber et al. 2021). However, ignoring the stochastic current fluctuations across the cell membrane in this approach hinders the prediction of the spiking efficiency seen in experiments (Fig. 1 and 3).

In order to show the impact of ion current fluctuations we are investigating the loudness discrimination with cochlear implants in a forthcoming study using a combined HH1 and HH10 model. As mentioned above, the diameter of an auditory nerve fiber is much smaller in its peripheral part than after the soma. As a consequence, the simulated RS is about five times larger if the implanted electrode is close to the fiber ending in comparison to a position close to the center of the cochlea where it elicits spikes in the thick axon. As loudness perception, coded by the spiking rates of the stimulated fibers, depends on the dynamic range, we predict better loudness discrimination for electrodes close to the fiber endings. This effect agrees with a study on intensity discrimination of 14 cochlear implant users with two types of electrode placements: subjects with the electrodes close to the cochlea center (far from fiber ending) had an average of only 9 discriminable intensity steps as compared to 23 steps in the other group (Kreft et al. 2004). Note that the classical nerve fiber model is useful for threshold calculations but is not of help to find the dependence of spiking rate on stimulus intensity.

(2) The figures should be improved, for example, the first letter should be capitalized. There are many errors in the References. Some recent references may be needed.
We improved the figures as suggested, corrected errors in the references and added recent references.

**Reviewer #2**: The problem of the paper comes from its idea. The authors consider that
1) the neuronal signal is binary, but there are many papers that subthreshold activity plays a significant role, see https://doi.org/10.1523/JNEUROSCI.19-24-10727.1999 for example (rather old paper!);
We agree that neural networks often generate oscillatory signals. As an example a stimulating pulse from a microelectrode in the retina can cause early and late responses recorded in the axon of a ganglion cell. The early spike is the consequence of the direct axon stimulation, the late from network activities. However, including non-axonal segments in our study overstrains the readership. Moreover, the individual electrical characteristics of somatic and dendritic membranes hinder a general treatment. Because of these facts we concentrated our investigation on the stimulations of axons and not on weak current stimulations of neurons in a network. To avoid confusion of the reader we did not discuss any neural network contributions to subthreshold activities.

2) the external input is considered to be Gaussian (the 1st reviewers also addressed this problem, but the authors could not answer properly), but there are papers considering that this is not the case, see https://doi.org/10.1103/PhysRevE.97.060302 as an example.
Our model is based on the report of Sigworth (1980) on the variance of sodium current fluctuation and on the common assumption of Gaussian noise in neural membranes. In order to test the sensitivity of our results we simulated the diameter dependence with an equally distributed noise and we found quite similar parameters. We added the following text.
The approximation quality to Verveen's formula is primarily determined by knoise and root of maximum sodium conductance times membrane area (Eq. 1), while the distribution of X seems to play only a minor role. Instead of assuming X to be normally distributed, let X now be uniformly distributed on the interval [-1,1].

For the same values of knoise, regression equations result under the assumption of uniform distribution with deviations in the coefficients in the order of 1%:

HH10, knoise = 0.00042:	log RS = - 1.53 – 0.77*log d	(12u)
CRRSS, knoise = 0.0038:	log RS = - 1.51 – 0.79*log d	(13u)
HH1, knoise = 0.0038:	log RS = - 1.47 – 0.82*log d	(14u)
